# DESCRIBING A CONCEPT OF NURSE INTUITION AND IMPLICATIONS FOR DEMENTIA CARE IN NURSING HOMES

**DOI:** 10.1093/geroni/igz038.3087

**Published:** 2019-11-08

**Authors:** Rebekah Perkins, Katherine Supiano’katherine supiano@hsc utah edu’ kather

**Affiliations:** University of Utah, Salt Lake City, Utah, United States

## Abstract

The purpose of this concept analysis was to explore nurse intuition historically and in current literature, appraise the value of nurse intuition to research and practice, and discuss implications for practice in caring for residents with behavioral and psychological symptoms of Dementia (BPSD) in nursing homes. To date, no research has examined the presence or utility of nurse intuition in the nursing home setting. A conceptual analysis using pragmatic utility was chosen and based on the work of Walker and Avant (2005). In the literature, nurse intuition is characterized by attributes of knowledge not preceded by inference, knowledge that is holistic in nature, independent of linear thinking, and drawn from synthesis instead of analysis. Nurse intuition is based on preconditions of experience, empathy, limited information, and limited time to make vital decisions about patient care. Studies on nurse intuition have been criticized for their lack of rigor and empirical evidence of the effect of nurse intuition on positive patient outcomes. The attributes of intuitive nursing practice have important implications in nursing applied to residents with BPSD. The nursing home nurse with extensive educational and experiential knowledge is well-situated to understand the complex, changing needs of residents exhibiting various forms of BPSD in an effort to communicate their needs. Future studies on nurse intuition should focus on early education in dementia care, nurse residency-mentor programs to enhance intuitive thinking in the management of BPSD, and more empirical studies on the use of intuition in the context of dementia care.

